# MRI and CT of anal carcinoma: a pictorial review

**DOI:** 10.1007/s13244-012-0199-3

**Published:** 2012-12-04

**Authors:** Massimo Tonolini, Roberto Bianco

**Affiliations:** Department of Radiology, “Luigi Sacco” University Hospital, Via G.B. Grassi 74, 20157 Milan, Italy

**Keywords:** Anal carcinoma, Anus, Human immunodeficiency virus, Chronic inflammatory bowel diseases, Computed Tomography (CT), Magnetic Resonance Imaging (MRI)

## Abstract

**Background:**

Squamocellular anal carcinoma is increasingly diagnosed in patients with risk factors.

**Methods:**

State-of-the-art imaging with magnetic resonance imaging (MRI) using phased-array coils and volumetric multidetector computed tomography (CT) provides detailed visualisation of anal disorders, identification and extent assessment of neoplastic tissue, detection and characterisation of nodal and visceral metastases. MRI has been recommended by the European Society for Medical Oncology (ESMO) as the preferred modality of choice to stage anal cancer, taking into account the maximum tumour diameter, invasion of adjacent structures and regional lymph node involvement.

**Results:**

Cross-sectional imaging techniques allow the identification of coexisting complications, and differentiation from other perineal abnormalities.

**Conclusion:**

Cross-sectional imaging is useful for planning radiotherapy, surgical drainage or salvage abdomino-perineal resection. After chemo-radiotherapy, MRI follow-up provides confident reassessment of therapeutic response, persistent or recurrent disease.

***Teaching Points*:**

• *Anal carcinoma is increasingly diagnosed in patients with human immunodeficiency virus (HIV), anoreceptive intercourse, chronic inflammatory bowel disease.*
• *An established association exists with human papillomavirus (HPV) infection and premalignant intra-epithelial dysplasia.*
• *Phased-array MRI is recommended as the preferred imaging modality for regional staging.*
• *Imaging allows detection of infectious complications, planning of radiotherapy or salvage surgery.*
• *Follow-up MRI allows reliable assessment of therapeutic response after chemo-radiotherapy.*

## Introduction

An uncommon malignancy in the general population, squamocellular anal carcinoma (SCAC) accounts for approximately 1 % of all gastrointestinal neoplasms and less than 5 % of anorectal tumours. In past decades, SCAC was usually diagnosed at a relatively advanced age with a significant female predominance, and believed to be an indolent disease secondary to chronic irritation. In recent years, similarly to uterine cervix dysplastic changes, oncogenic human papillomavirus (HPV) has been detected in the vast majority (up to 90 %) of invasive SCACs, and linked to the development of low- and high-grade premalignant anal intra-epithelial neoplasms (AIN), particularly with high-risk or multiple HPV serotypes infection [1, 2].

Furthermore, the incidence of SCAC is steadily increasing, particularly in patients with risk factors such as human immunodeficiency virus (HIV) infection, history of anoreceptive intercourse, coexistent cervical dysplasia or cancer, immunosuppression, inflammatory bowel diseases (IBD) and cigarette smoking. Currently, at least half of SCACs occur in relatively young (40–60 years) HIV-positive individuals, most often men who have sex with men (MSM) [3–5].

## Regional anatomy and imaging techniques

The surgical anus is about 4 cm long from the anorectal junction to the perianal skin on the external anal margin (verge). The internal anal sphincter consisting of smooth muscle is separated from the external, striated muscle sphincter by the fatty intersphincteric space. Along with the puborectalis and levator ani muscles, the external anal sphincter forms the sphincter complex. Located approximately halfway along the anus, the dentate line marks the transition from the squamous epithelium to the intestinal mucosa. Thus, histologically SCACs can be either keratinising or non-keratinising according to their origin below or above the dentate line, although with similar biological behaviour [6, 7].

Lymphatic drainage of anal neoplasms varies according to the primary lesion site. Anal margin and anal canal SCAC originating distal to the dentate line drain to the inguinal and femoral lymph nodes. When the primary tumour arises above the dentate line, regional lymph nodes include the inguinal, internal iliac and perirectal nodes, whereas the external, common iliac and para-aortic nodes are considered non-regional [6–8].

Because of its anatomical location, in most cases SCAC is diagnosed clinically in patients with rectal bleeding, pain, discharge or palpable masses. Alternatively, lesions may be detected during follow-up of high-risk individuals. Following physical examination including digital rectal and vaginal examination, ano-proctoscopy and biopsy, imaging is required to evaluate the local extent of the lesion, lymph node involvement, possible invasion of adjacent organs and distant metastases [6, 9].

Imaging the anal canal and perianal structures may prove technically challenging to perform and interpret. In past years, trans-anal ultrasound (TRUS) and magnetic resonance imaging (MRI) techniques allowed an accurate assessment of tumour size and depth of mural invasion [7, 10, 11]. Unfortunately, in patients with anal lesions, positioning of endoanal sonography probes and MRI coils is hampered by pain and stricture. Trans-anal imaging combines an excellent spatial detail with a limited field-of-view that prevents panoramic assessment of entire ischiorectal spaces and of regional lymph nodes. Furthermore, TRUS has limited specificity for differentiation of residual tumour versus post-treatment fibrosis [11–13].

Currently, MRI performed using external phased-array coils on high-magnetic-field scanners is the imaging modality of choice to investigate the anal region. Significant advantages of MRI include its native multiplanar capability, superior soft-tissue differentiation, biological non-invasiveness and optimal safety profile of gadolinium-based contrast agents. No special patient preparation is needed. Acquisition protocols heavily rely on high-resolution T2-weighted sequences along three planes, with coronal and axial scans planned slightly oblique, respectively parallel and perpendicular to the long axis of the anal canal. Despite the increased tumour conspicuity provided by background fat suppression, short-tau inversion recovery (STIR) sequences are less useful because of limited spatial detail and difficulty to delineate anatomic landmarks. At our Centre, T1-weighted sequences including fat suppression in at least one plane are routinely acquired following standard-dose intravenous gadolinium contrast, to allow detection of lesion enhancement. Conversely, other authors discourage post-contrast MRI acquisitions by stating that enhanced images do not offer additional information to the high soft tissue contrast intrinsic to T2-weighted imaging [7, 10, 13–15].

Although with limited contrast resolution compared with MRI, volumetric multidetector computed tomography (MDCT) acquisitions including image reformations along arbitrary planes allow visualisation of anorectal abnormalities in their cranio-caudal extent with relationship to key anatomical landmarks such as the sphincter complex [16–18].

## Imaging features and tumour staging

MRI provides a detailed visualisation of the anal canal and nearby anatomical structures. Although the dentate line is not directly recognisable, its position can be inferred as it corresponds approximately to the upper portion of external sphincter muscles. Sensitivity of TRUS and MRI for the identification of SCAC has been reported to approach 90–100 %, with high concordance regarding tumour size, although on a limited number of patients, and more precise results with ultrasound for smaller, superficial tumours [19].

**Fig. 1:**
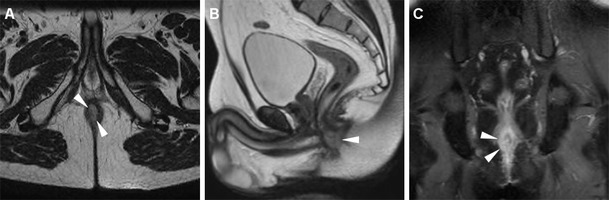
A 40-year-old MSM with bioptic diagnosis of SCAC. Axial (**a**) and sagittal (**b**) T2-weighted images show 2-cm hyperintense nodule contained within the internal sphincter muscle, intensely enhancing as seen on post-contrast fat-suppressed coronal T1-weighted image (**c**), consistent with T1 tumour (*arrowheads*)

**Fig. 2:**
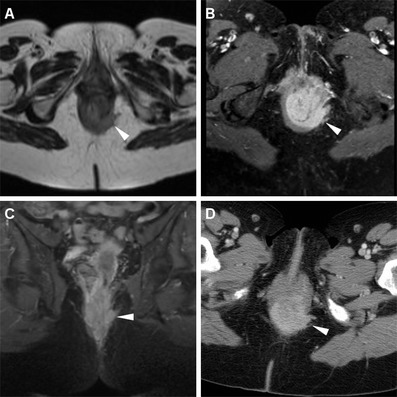
A 62-year-old female with biopsy-proven SCAC. Axial T2-weighted (**a**), post-contrast fat-suppressed axial (**b**) and coronal (**c**) T1-weighted images, and corresponding enhanced image from body CT (**d**) show a 5.5-cm long (T3) enhancing tumour with infiltration of the left ischioanal fatty space (*arrowheads*)

**Fig. 3:**
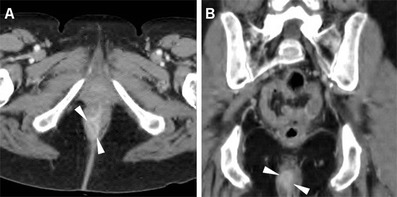
A 57-year-old woman undergoing abdomino-pelvic MDCT for unrelated reasons. Post-contrast axial (**a**) and coronal reformatted (**b**) detailed images of the anorectal region identify an unexpected 2-cm right-sided enhancing anal nodule. Subsequent clinical and bioptic assessment confirmed poorly symptomatic ulcerated SCAC

**Fig. 4:**
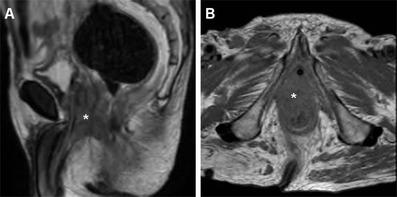
An elderly, 92-year-old man with previous prostatectomy and kidney failure has unenhanced MRI. Sagittal T2- (**a**) and axial T1-weighted (**b**) images show 5-cm long solid, inhomogeneous neoplastic tissue (*) extending from the anus to encase the proximal urethra (note catheter in place)

Neoplastic tissue in the anal canal has low-to-intermediate T1 signal intensity and positive enhancement after intravenous gadolinium contrast. On T2-weighted and STIR sequences, untreated neoplasms display intermediate signal intensity, lower to that of normal ischioanal fat and almost always superior to the internal reference standard represented by uninvolved anal sphincters and gluteal muscles (Figs. 1, 2). Although with limited sensitivity compared with MRI, on CT images SCAC may be detected as solid, enhancing nodules or masses within the anus (Figs. 2, 3). Progressive heterogeneity is observed in larger lesions (Fig. 4) [7, 9, 10, 13].

Staging is essential for both prognostic information and correct therapeutic planning, and is performed according to the UICC/AJCC tumour-node-metastasis (TNM) system (Table 1), including local lesion extent, lymph node status and distant metastatic spread. In 2010 the European Society for Medical Oncology (ESMO) recommended MRI as the primary imaging modality to accurately stage SCAC, taking into account the maximum tumour diameter, possible invasion of adjacent organs and nodal involvement [6, 13].

**Table 1:** Tumour-node-metastasis (TNM) staging of anal carcinoma according to lesion site of origin

	Anal canal	Anal margin
Primary tumour (T)		
TX		Primary tumour cannot be assessed
T0		No evidence of primary tumour
T1		Tumour ≤2 cm in greatest dimension
T2		Tumour 2–5 cm in greatest dimension
T3		Tumour >5 cm in greatest dimension
T4	Tumour of any size invading adjacent organ(s) e.g. vagina, urethra, bladder	Tumour invading deeper structures (skeletal muscle, cartilage)
Regional lymph nodes (N)		
NX		Regional lymph nodes cannot be assessed
N0		No regional lymph node metastasis
N1	Metastasis in perirectal lymph node(s)	Regional lymph node metastasis
N2	Metastasis in unilateral internal iliac and/or inguinal lymph node(s)	
N3	Metastasis in internal iliac and perirectal lymph nodes and/or bilateral internal iliac and/or bilateral inguinal lymph nodes	
Distant metastasis (M)		
M0		No distant metastasis
M1		Distant metastasis

Particular care should be applied to choosing the longest lesion diameter on T2-weighted images, since correct T parameter staging relies on this measure being below 2 cm, over 5 cm or intermediate (Fig. 1) [7, 9, 10, 13].

**Fig. 5:**
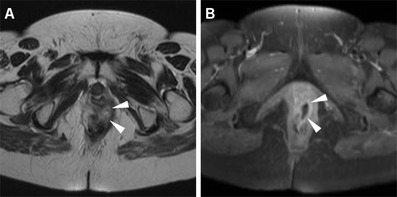
A 32-year-old HIV-positive woman with clinical diagnosis of anovaginal fistula. Axial T2-weighted (**a**) and post-contrast fat-suppressed axial T1-weighted (**b**) images show inhomogeneous anal tissue invading the left aspect of the vagina (*arrowheads*), with internal non-enhancing necrosis and peripheral enhancement. Biopsy diagnosed SCAC with superimposed infection

MRI demonstrates good correlation with physical findings concerning T stage, whereas infiltration of adjacent organs and sometimes tumour size are clinically underestimated. Extramural neoplastic spread may involve the sphincter complex muscles (external sphincter, levator ani and puborectalis) and most commonly occurs towards the anterior urogenital triangle with possible vaginal, urethral or bladder involvement. Sometimes, the tumour may also extend laterally with invasion of the ischioanal fossa (Fig. 2), superiorly to the rectum and mesorectal compartment, or inferiorly to the skin and subcutaneous planes of the perianal region. In such instances, T2-hyperintense solid tissue is seen infiltrating the even more hyperintense fat in the ischiorectal (Fig. 2) and subcutaneous spaces, encasing the lower signal intensity skeletal muscles, or isointense structures such as the vagina (Fig. 5), prostate and urethra (Fig. 4). Notably, radiologists should remember that anal canal carcinoma directly invading the rectal wall, perianal skin, subcutaneous or the sphincter muscle does not imply assessing the tumour stage as T4. For anal margin SCAC, a T4 lesion is defined by invasion of deeper structures such as the skeletal muscle or cartilage [9, 10, 13].

**Fig. 6:**
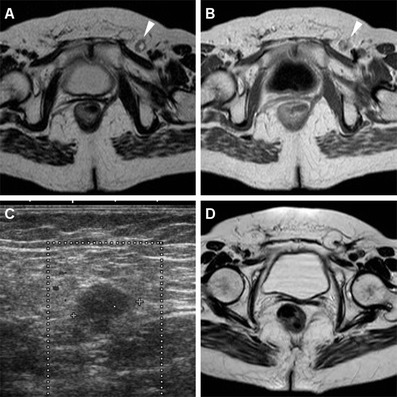
A 65-year-old woman with history of previously treated small SCAC 3 years earlier. Axial T2- (**a**) and post-contrast T1-weighted (**b**) images show roundish 1-cm left inguinal node (*arrowheads*) with internal fluid-like necrosis and inhomogeneous enhancement, confirmed by ultrasound (**c**) as hypoechoic with loss of normal nodal structure. Surgical exeresis (postoperative status as seen in **d**, follow-up MRI) confirmed metastatic node from SCAC

**Fig. 7:**
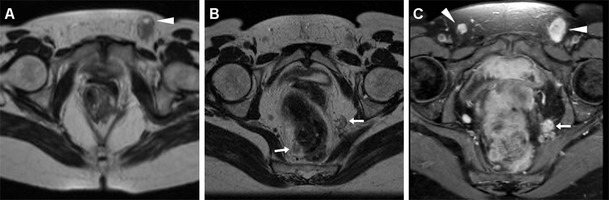
The same patient as in Fig. 2. Axial T2-weighted (**a**,**b**) and post-contrast fat-suppressed T1-weighted (**c**) images show inguinal nodal metastases, larger on left side (*arrowheads*) plus bilateral enhancing perirectal adenopathies (*arrows*)

The incidence of regional nodal involvement increases with primary tumour size. Lymph node metastases may be present (in 25 % of cases) even with superficial (up to T2) SCACs, and are unreliably assessed clinically. Nodal staging evaluation relies on the distance from the primary tumour rather than on the number of involved nodes [6]. MRI is highly helpful to assess lymph node metastatic involvement, although the mere size criterion is far from accurate and associated with both false-positive and false-negative results. Short-axis threshold values of 8 mm, 5 mm and 10 mm have been suggested for pelvic, perirectal and inguinal lymph nodes, respectively. Additional helpful features to increase specificity include loss of the normal bean-shaped morphology and fatty hilum, internal T1 and T2 signal heterogeneity with central necrosis, and inhomogeneous enhancement (Figs. 6, 7) [10, 13].

As suggested by ECCO guidelines, search for distant spread is usually performed by means of contrast-enhanced body MDCT, with conventional imaging appearances of liver and lung metastases. Dissemination is very uncommon (less than 5 % of patients at initial diagnosis, and is usually encountered in association with post-treatment recurrence [20]. Alternatively, in immunocompetent patients ^18^F-fluorodeoxygluocose positron emission tomography (FDG-PET/CT) has high specificity for nodal and visceral dissemination. At diagnosis, FDG-PET/CT may alter staging of anal SCAC in 20 % of patients, leading to inclusion of involved pelvic or inguinal lymph nodes in the radiation field [6, 7].

## Complications

**Fig. 8:**
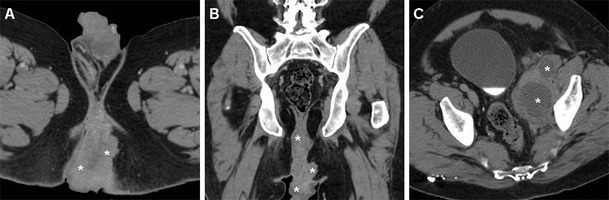
A 60-year-old man with AIDS and clinical finding of ulcero-fungating anal mass. Axial (**a**) and coronal reformatted (**b**) CT images show moderately heterogeneous tissue (*) in its entire longitudinal extent from the anorectal junction to below the anal verge, associated with large necrotic iliac adenopathies (* in **c**). Biopsy confirmed superinfected SCAC

**Fig. 9:**
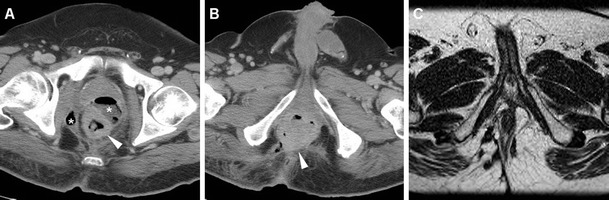
A 53-year-old man with purulent drainage and clinical diagnosis of perianal inflammation. Axial images (**b**,**c)** from urgent contrast-enhanced MDCT show solid circumferential thickening of the anal canal (*arrowheads*) associated with abscess collections with mixed gas-fluid content (*) and fistulas crossing the ischioanal space. Surgical examination under anaesthesia including biopsies revealed ulcerated SCAC with superinfection. After surgical drainage and subsequent chemo-radiotherapy, MRI (**c**) shows complete resolution of both inflammatory and neoplastic changes with residual T2-hypointense fibrotic tracks

**Fig. 10:**
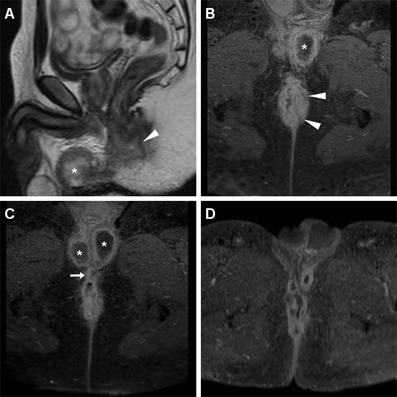
A 39-year-old HIV-infected man with biopsy-proven SCAC. Staging MRI confirms left-sided anal thickening (*arrowheads*) with abnormal T2 signal intensity (**a**) and strong contrast enhancement (**b**). Incidentally, two abscess collections with necrotic content and peripheral enhancement are seen ventrally, connected to the anal canal by a fistulous track (*arrow* in **c**). Subtotal regression of changes after treatment is seen on follow-up MRI (**d**)

In our experience, not infrequently anal tumours coexist with inflammatory conditions such as proctitis and abscesses. In such instances, MDCT and MRI provide confident detection of perirectal inflammatory changes and purulent collections that are differentiated from solid neoplastic tissue, thus allowing a correct therapeutic choice including surgical drainage as necessary (Figs. 5, 8, 9, 10). Resolution of associated inflammatory changes during treatment is easily monitored by cross-sectional imaging (Figs. 9, 10) [15, 21].

Furthermore, cross-sectional imaging particularly with MRI also proves useful to differentiate anal carcinoma from other causes of local pain and perineal masses, such as pilonidal sinus diseases, Gartner duct or Bartolini gland cysts, tailgut cysts, uncommon soft-tissue neoplasms, urethral cancer, lymphoma or metastases [14, 22].

## Treatment

In the past, SCAC was treated with abdomino-perineal resection and permanent colostomy. Currently, anal margin and small canal tumours without evidence of nodal spread may be successfully excised. In all other cases, the standard treatment includes radiation combined with mitomycin-C plus infusional 5-FU chemotherapy, yielding an 80 % 5-year survival rate with preservation of sphincter function. Advanced T3/T4 tumours have worse outcomes with a 40–68 % 3-year disease-free survival. New neoadjuvant and adjuvant drugs are being investigated to treat advanced disease. Salvage surgery with abdomino-perineal resection is reserved for persistent or recurrent tumours [3, 6, 23–25].

**Fig. 11:**
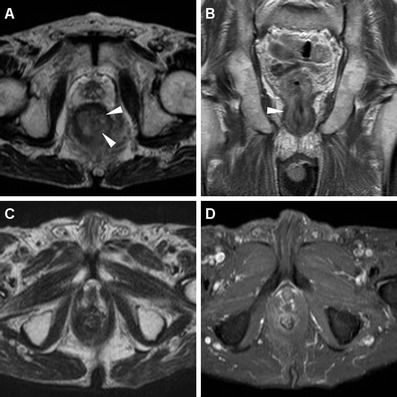
An 83-year-old man with known SCAC. Initial MRI shows moderate circumferential thickening of the anus with T2-hyperintense signal (**a**) and contrast enhancement (**b**) (*arrowheads*). Complete disappearance of the lesion is observed on axial T2 (**c**) and post-contrast fat-suppressed T1-weighted (**d**) images following chemo-radiotherapy

**Fig. 12:**
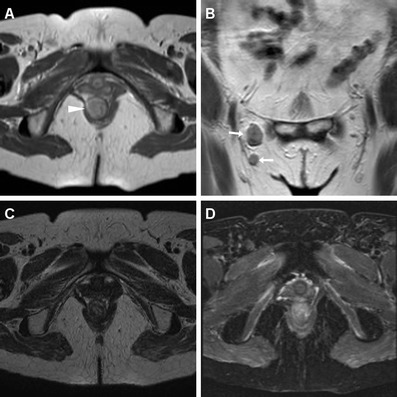
A 46-year-old female patient with biopsy-proven SCAC. MRI staging including axial (**a**) and coronal (**b**) T2-weighted images detect a solid, 3-cm eccentric anal mass (*arrowhead*) consistent with T2 tumour, associated with right inguinal adenopathies with analogous signal features. After chemo-radiotherapy, MRI follow-up (**c**, **d**) shows complete disappearance of both anal neoplastic solid tissue and lymphadenopathies

**Fig. 13:**
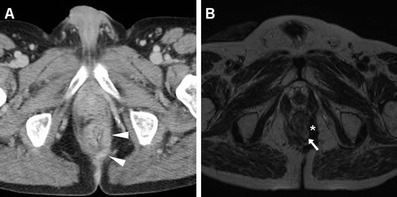
A 46-year-old HIV-infected man with biopsy-proven SCAC. Initially, MDCT (**a**) shows left-sided thickening with involvement of the external sphincter (*arrowheads*). After chemo-radiotherapy, lesion regression is observed on MRI with appearance of T2-hypointense fibrosis (*) and hyperintense nodule (*arrow*) consistent with residual tumour

**Fig. 14:**
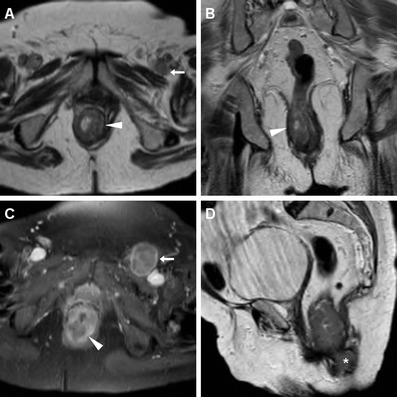
An elderly, 85-year-old lady with biopsy-proven SCAC and multiple comorbidities. Initial MRI (**a**,**b**) shows showed circumferential anal wall thickening with abnormal solid signal (*arrowheads*) measuring 6 cm in length, plus a suspicious centimetric left inguinal lymph node (*arrow* in **a**), findings consistent with T3N2 lesion. After reduced chemo-radiotherapy, follow-up MRI (**c**, **d**) 4 months later disclosed progression and partial necrosis of both primary tumour (*arrowhead*) and inguinal adenopathy (*arrow*), plus appearance of an exophytic tissue mass protruding from the external anal orifice (* in **d**)

After radio-chemotherapy, imaging follow-up with MRI represents a useful complement to clinical evaluation in the assessment of therapeutic response. Shortly after treatment completion, interpretation of MRI is usually challenging due to the superimposition of inflammatory changes resulting from radiotherapy. Performed at least 6–8 weeks after treatment completion, MRI provides confident, reproducible assessment of post-treatment modifications. Findings indicative of a positive response include size reduction and diminished T2 signal intensity of the treated tumour and associated adenopathies (Figs. 10, 11, 12). The appearance of T2-hypointense signal is consistent with fibrosis, although it does not allow excluding minor residual neoplastic foci for sure (Fig. 13). Size decrease usually becomes evident more than 6 months following treatment. Stability in size and signal intensity of any residual abnormality visible at MRI in the site of the treated lesion 1 year after therapy has been reported to be strongly associated with a favourable outcome. Locoregional and/or distant recurrence occurs in up to 35 % of treated patients, and is strongly associated with advanced (T3–T4) stage and nodal involvement at presentation [7, 20]. Persistent and locally recurrent tumours often display an aggressive behaviour, with possible extensive invasion of the adjacent organs and pelvic bony structures, and a tendency for lymphatic dissemination (Figs. 14) [9, 10, 13].

## HIV-associated anal carcinoma

After the introduction of highly active anti-retroviral treatment (HAART), people with HIV infection or acquired immunodeficiency syndrome (AIDS) gained a greatly improved life expectancy with better immune conditions, at the price of an increased tendency to develop tumours. Although it is considered a non-AIDS-defining malignancy, currently SCAC ranks third (8.2 %) among neoplasms observed in HIV/AIDS populations, with a substantially higher incidence in MSMs and in long-standing infected people [4, 26].

Dysplastic intraepithelial lesions are highly prevalent in HIV-infected people, and HIV represents a marker for coinfection with other sexually transmitted diseases such as HPV. Since the risk of developing SCAC increases with the total time elapsed with CD4+ count below 200 cells/μl, it has been hypothesised that HIV-related immune suppression acts as a cofactor to HPV in the development of anal dysplasia and progression to overt carcinoma [1, 3, 4].

Screening procedures including high-resolution anoscopy and cytology smears are increasingly adopted at HIV care centres, to allow detection of SCAC precursors and early-stage tumours amenable to limited excision and topical therapies. Prevention should limit the occurrence of advanced stages at diagnosis in the future [4, 26–29].

A high prevalence (at least 30 %) of anorectal complaints is characteristic of HIV-positive patients, particularly those practicing anoreceptive intercourse. Differential diagnosis encompasses a wide spectrum of abnormalities, including non-specific anal diseases such as haemorrhoids, fissures, fistulas and abscesses, along with venereal infections, viral ulcers, and a non-negligible (7 %) rate of neoplasms such as SCAC, lymphoma and Kaposi’s sarcoma. Therefore, when performing cross-sectional imaging procedures on HIV-infected patients, even for unrelated complaints, special attention should be paid to the anal region, with a focus on the possible identification of solid, enhancing tissue consistent with tumour that indicates need for biopsy (Figs. 5, 8, 10, 13) [21, 30, 31].

Despite initial discouraging reports, in the HAART era HIV-positive patients are likewise treated with standard chemo-radiation regimens, reaching satisfactory results in terms of local control and survival rates, although with increased toxicity and frequent local recurrences [4, 5, 26, 27, 32].

## Anal carcinoma in inflammatory bowel diseases

**Fig. 15:**
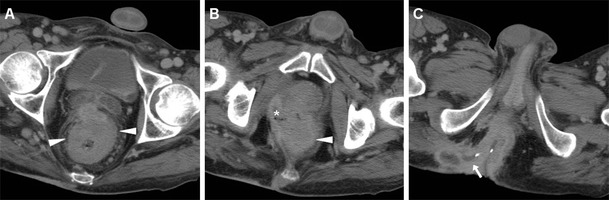
A 40-year-old male patient with long-standing perianal Crohn’s disease, being treated with seton. Contrast-enhanced MDCT images (**a**, **b**, **c** in cranio-caudal order) show right-sided levator ani abscess (*), extensive perianal fistulisation occupying the ischioanal space (*arrow*), and marked solid-appearing circumferential anorectal thickening (*arrowheads*). Abdomino-perineal resection for SCAC was performed

**Fig. 16:**
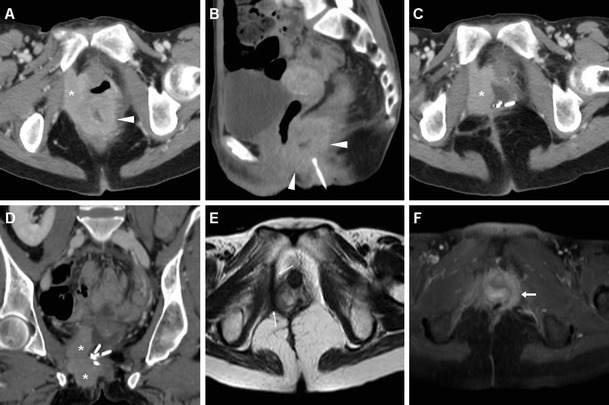
A 39-year-old woman with history of ulcerative colitis and perianal inflammation. Initial multiplanar MDCT (**a**, **b**) identified enhancing anal thickening (*arrowheads*) with right-sided vaginal infiltration and solid tissue (*) extending to reach the internal obturator muscle. After biopsy confirmation of SCAC and surgical debulking with colostomy, repeat MDCT (**c**, **d**) detected enlarging neoplastic residue (*). Shortly after chemo-radiotherapy, MRI (**e**) detected the formation of a thick hypointense fibrotic band in the site of the regressed tumour (*thin arrows*). MRI follow-up (**e**) identified appearance of a contralateral enhancing tissue band interpreted as suspicious for local recurrence (*arrow*). After negative clinical reassessment and PET findings, this post-treatment finding remained stable on further MRI studies (not shown)

Some literature reports have highlighted the increased risk of anus and lower rectum carcinomas associated with long-standing, severe perianal fistulising Crohn’s disease (CD) (Fig. 15). In patients with CD, SCAC reaches a 14 % proportion among all colorectal cancers, which is ten-times higher than the usual figure. Furthermore, CD patients develop anorectal carcinomas at a younger age (20 years earlier) than the general population. According to the hypothesised pathogenesis, fistulas probably allow HPV an easier access to the epithelial layers, and chronic mucosal regeneration may ultimately lead to neoplastic changes. However, anal tumours may occasionally develop in patients with ulcerative colitis (UC)-related (Fig. 16) or cryptogenetic chronic perianal inflammation [33, 34].

Diagnosis is often unsuspected or delayed because of pre-existent, unspecific complaints and clinical assessment is hampered by complex inflammation with stricture and local pain. As a result, IBD-associated anal cancers are often advanced at presentation, may require extensive surgery plus chemotherapy and radiotherapy, and are associated with a severe prognosis [34]. Therefore, patients with early-onset or long-standing perianal CD should undergo clinical and imaging surveillance, particularly when new or changed symptoms develop. Radiologists should be aware of the increased risk for anorectal cancer in middle-aged IBD patients, and clearly report any solid tissue as suspicious for neoplasm and suggest biopsy (Figs. 15, 16) [33–35].

## Conclusion

The established association with HPV infection and premalignant intra-epithelial dysplastic changes provides insight into the pathogenesis of HIV- and IBD-related anal cancers, and the possibility of prevention or early diagnosis through screening of high-risk individuals [1, 2].

State-of-the-art cross-sectional imaging with high-resolution MRI using external phased-array coils and multiplanar MDCT allow detailed, comprehensive visualisation of abnormalities involving the anus and perineal region. Currently, MRI represents the modality of choice for primary regional staging of SCAC, assessment of complications, of therapeutic response following chemo-radiotherapy, and of possible recurrences [9, 10, 13].
