# Erratum to: MRI and CT of anal carcinoma: a pictorial review

**DOI:** 10.1007/s13244-013-0221-4

**Published:** 2013-01-30

**Authors:** Massimo Tonolini, Roberto Bianco

**Affiliations:** Department of Radiology, “Luigi Sacco” University Hospital, Via G.B. Grassi 74, 20157 Milan, Italy

**Erratum to: Insights Imaging**

**DOI 10.1007/s13244-012-0199-3**

In this article, Table 1 has been published in a confusing layout. We would like to clarify that the table should look as follows:

**Table 1:**
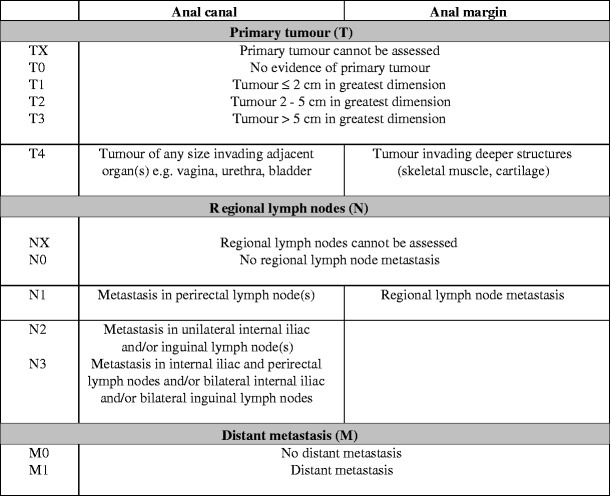
Tumour-node-metastasis (TNM) staging of anal carcinoma according to lesion site of origin

